# Work–Family Conflict and Self-Rated Health: the Role of Gender and Educational Level. Baseline Data from the Brazilian Longitudinal Study of Adult Health (ELSA-Brasil)

**DOI:** 10.1007/s12529-015-9523-x

**Published:** 2015-11-23

**Authors:** Rosane Härter Griep, Susanna Toivanen, Cornelia van Diepen, Joanna M. N. Guimarães, Lidyane V. Camelo, Leidjaira Lopes Juvanhol, Estela M. Aquino, Dóra Chor

**Affiliations:** Laboratory of Health and Environment Education, Oswaldo Cruz Institute, Oswaldo Cruz Foundation, Avenida Brasil, 4365, Manguinhos Rio de Janeiro, 21040-360 Brazil; Centre for Health Equity Studies, Stockholm University and Karolinska Institutet, Stockholm, Sweden; Department of Geography, Portsmouth University, Portsmouth, UK; National School of Public Health, Oswaldo Cruz Foundation, Rio de Janeiro, Rio de Janeiro Brazil; Postgraduate Program in Public Health, Faculty of Medicine, Universidade Federal de Minas Gerais, Minas Gerais, Brazil; Institute of Collective Health, Federal University of Bahia, Salvador, Bahia Brazil

**Keywords:** Gender, Work and family conflict, Self-rated health, Educational level, ELSA-Brasil cohort study

## Abstract

**Purpose:**

This study examined gender differences in the association between work–family conflict and self-rated health and evaluated the effect of educational attainment.

**Method:**

We used baseline data from ELSA-Brasil, a cohort study of civil servants from six Brazilian state capitals. Our samples included 12,017 active workers aged 34–72 years. Work–family conflict was measured by four indicators measuring effects of work on family, effects of family in work and lack of time for leisure and personal care.

**Results:**

Women experienced more frequent work–family conflict, but in both genders, increased work–family conflict directly correlated with poorer self-rated health. Women’s educational level interacted with three work–family conflict indicators. For time-based effects of work on family, highly educated women had higher odds of suboptimal self-rated health (OR = 1.54; 95 % CI = 1.19–1.99) than less educated women (OR = 1.14; 95 % CI = 0.92–1.42). For strain-based effects of work on family, women with higher and lower education levels had OR = 1.91 (95 % CI 1.48–2.47) and OR = 1.40 (95 % CI 1.12–1.75), respectively. For lack of time for leisure and personal care, women with higher and lower education levels had OR = 2.60 (95 % CI = 1.95–3.47) and OR = 1.11 (95 % CI = 0.90–1.38), respectively.

**Conclusion:**

Women’s education level affects the relationship between work–family conflict and self-rated health. The results may contribute to prevention activities.

## Introduction

With greater participation of women in the workforce over recent decades in most parts of the world, both men and women are involved in work and family life [1, 2]. In Brazil, women have only relatively recently entered the labour market, but their contribution has grown rapidly. The proportion of women in the workforce increased from 32 % in 1980 to 57 % in 2009. The female-to-male labour force participation rate also increased from 52.2 % in 1990 to 73.3 % in 2010 and has continued to increase steadily [3, 4]. This is good for the emancipation and economic independence of women but can also have its drawbacks as women and men now need to balance their responsibilities at home and work.

The division of household tasks and childcare responsibility between working men and women, however, has not undergone similar changes and is still unequal [5]. It also varies by country and region [6]. For example, in Sweden, a country with high gender equality, women carry out an average of 26 h of unpaid work each week, whereas men do about 21 h [7]. In Europe as a whole, women spend an average of 26 h per week on care and household activities, compared with 9 h for men (European Commission, Report on Progress on equality between women and men in 2013). In Brazil, the average unpaid work per week is 25 h for women and about 10 h for men [3]. Although men have slightly increased their participation in housework and childcare in Brazil, women still perform most family tasks and spend more time on unpaid domestic work even if they are in full-time paid work [1, 3, 8–11].

Balancing work and family demands is challenging, and one or other may require more time and attention than is available. The work–family conflict is defined as ‘a form of interrole conflict in which the role pressures from the work and family domains are mutually incompatible in some respect’ [12]. This imbalance is also conceptualized as work–life conflict or work–life imbalance [13, 14]. Although correlated, work–family conflict and work–life conflict measures are, however, different [15]. The work–family conflict is more related to the lack of boundaries between work and family spheres and could be moderated by family status. Work–life imbalance research focuses on the spillover effect in a broader context, in which work influences experiences in the non-work sphere (for example, time for leisure, friends and family life) [15]. In the present study, we focused on work–family boundary management (rather than broader work–life issues) in two basic directions: work-to-family or family-to-work conflict [16–22]. Most previous studies have focused on work-to-family conflict, viewed as resulting from occupational conditions [17, 19, 20, 22, 23]. Less often, family-to-work conflict has been investigated, and is viewed as arising from home and life circumstances [17–19, 24]. Some authors have postulated that family-to-work conflict could have more long-term consequences than work-to-family conflict [25, 26] and also have a greater influence on women’s health [22, 26]. Other studies have also discussed work-to-family conflict as being more detrimental to women’s health than to men’s health [27, 28]. A few studies have described characteristics of work–family conflict as two distinguishable forms: time-based (time devoted to one role makes it difficult to participate in another) and strain-based (excessive effort to perform in one domain affects performance in the other) [19, 22]. An additional form of conflict, behaviour-based work–family conflict, refers to specific behaviours in one role being incompatible with behaviours in the other [17, 19, 21]. However, little is known about gender-based antecedents or outcomes for each of these forms [17, 19, 22].

More recently, some authors have suggested that work and non-work are no longer separate domains and can simultaneously affect quality of life, leisure and health, with a different pattern according to gender [2, 18–20, 24, 29]. Based on this literature, we included a measure in the present study of both domains (work and family) simultaneously affecting leisure time and self-care.

Previous studies have investigated the association between work–family conflict and health status, such as common symptoms, mental health or depression [16, 19, 30–32], and whether work–family conflict may reduce the well-being benefits of employment [19, 33]. Some studies have also reported an association between work–family conflict and poorer self-rated health [16, 34–36]. Self-rated health expresses subjective as well as objective aspects of health and could reflect gender differences in stress response. It has been shown to be a predictor of future morbidity and mortality, functional decline and disability and higher utilization of health care [37–39].

Much of the literature shows that gender differences in work environment and family characteristics affect the association between work–family conflict and health [19, 27, 31, 34]. Gender is an essential determinant of inequalities in work–family conflict [36]. Most previous studies have distinguished between genders, and the results generally show a higher prevalence of work–family conflict and suboptimal self-rated health among women [36, 40, 41], although some studies found similar results in men and women [27, 32, 34].

Educational level also affects the experience of work–family conflict and the resulting mental health consequences [23, 42]. Individuals with higher educational attainment tend to express more work–family conflict. This might be due to high-pressure jobs and working longer hours than people with lower levels of education [23]. Educational level is also closely related to inequalities in health and is often used as a proxy for occupational prestige [23]. Groups with lower levels of education have a higher risk of mortality [43] and worse self-rated health than highly educated groups [44]. It is therefore possible that education level might modify the association between work–family conflict and self-rated health. To our knowledge, no previous studies have investigated this interaction considering gender stratification.

The association between work–family conflict and health status has been well-studied, mostly in western Europe and North America. In Brazil, the most populous country in South America, income and gender inequality remain high [3], but studies about influence of work–family conflict on health are scarce. This study therefore aims to investigate gender differences in the association between work–family conflict and self-rated health, and to evaluate whether educational attainment modifies this association, using data from the baseline of the Brazilian Longitudinal Study of Adult Health (ELSA-Brasil).

## Methods

### Study Design

The ELSA-Brasil design and concepts have been detailed elsewhere [45]. The study population consisted of 15,105 civil servants (46 % men, with ages ranging from 34 to 75 years). The ELSA-Brasil cohort comprised voluntary participants. Effort was made to recruit similar proportions of both genders, as well as predefined proportions of specific age groups and distinct occupational categories, to permit a wide socioeconomic gradient across the sample. Participants were recruited from five universities and one research institute in six Brazilian state capitals (Federal Universities of Bahia, Espírito Santo, Minas Gerais, and Rio Grande do Sul, the University of São Paulo, and the Oswaldo Cruz Foundation).

Baseline assessment (2008–2010) included comprehensive questionnaire interviews conducted by trained personnel and clinical and laboratory measurements [45–47]. The population in our study included only active workers (*n* = 12,096) and missing values on these central variables were excluded (*n* = 79). This resulted in a sample of 12,017 valid subjects (99.3 %) for analysis.

### Self-Rated Health

Self-rated health was evaluated by a single question: In general, compared with other people your age, how would you rate your health? The respondents answered on a five-point scale, later dichotomized into ‘good’ (good and very good) and ‘suboptimal’ (fair, poor and very poor).

### Work–Family Conflict

Four indicators of work–family conflict were measured. The three first items were based on the model designed by Frone and colleagues [16, 25], and the psychometric properties have been previously shown [48]. The first statement, assessing time-based work-to-family conflict, stated: *Demands (requirements or requests) from work keep you from spending the amount of time you would like with your family* (kappa = 0.63; confidence interval [CI] 0.52–0.71). The second statement, assessing strain-based work-to-family conflict, stated: *Demands (requirements or requests) from work make it difficult to fulfil domestic responsibilities, such as caring for the house and children* (kappa = 0.56; CI 0.45–0.67). The third statement, assessing family-to-work conflict, stated: *Demands (requirements or requests) from your family interfere with your responsibilities at work, such as getting to work on time, accomplishing daily tasks, travelling for work, and attending meetings outside the regular work schedule* (kappa = 0.46; CI 0.32–0.58). The fourth statement was designed by ELSA-Brasil researchers to cover the simultaneous effects of both work and family on lack of time for leisure and self-care [29]: *Demands (requirements or requests) from your family and work keep you from spending the amount of time you would like on your own care and leisure activities* (kappa = 0.70; CI 0.63–0.77). Each statement used a five-point frequency-based response scale: never to almost never, rarely, sometimes, frequently and very frequently. The categories were grouped into three levels: 1 was ‘never to rarely’ (reference category), 2 was ‘sometimes’ and 3 ‘frequently’.

### Covariates

The covariates examined were the following: age (continuous); education (‘lower’ lower than complete university degree or ‘higher’ complete university degree or above); marital status (married or living together; divorced, separated or widowed; or single living without partner); the presence of children under the age of five in the household (yes/no); the presence of a maid in the household (yes/no); hours worked per week (continuous); shift work (daytime employment without weekends, dayshifts which included weekends, and mostly nightshifts or mixed shifts); and self-reported medical diagnosis of chronic diseases (if respondents reported at least one of the following diseases, they were coded as ‘yes’, otherwise ‘no’ hypertension, diabetes, myocardial infarction, stroke and heart failure).

### Statistical Analyses

We performed all analyses separately for women and men. Chi-squared and *t* tests were used for descriptive analyses of covariates and work–family conflict. All covariates associated with the outcome (*p* ≤ 0.05) were included in multiple logistic regression analysis to estimate odds ratios (OR) and 95 % CI. The collinearity between the covariates was tested by generalized variance inflation factor (GVIF) [49]. Because of the risk of over-adjusting the four areas of work–family conflict, all covariates were tested individually to determine their effect on the association between each area of work–family conflict and self-rated health. To evaluate the importance of each variable in the model, we used deviance statistics and the Akaike information criterion (AIC). Lower values were considered a better fit.

Multiplicative interaction between work–family conflict indicators and education was assessed by the inclusion of an interaction term in the full adjusted regression models (work–family conflict*education). When the interaction term was significant (*p* < 0.1), the OR was estimated again with the effect of the interaction between the work–family conflict indicator and education [50].

All analyses were conducted in SPSS 22.0 for Windows and free software R, version 3.1.2 (R Development Core Team, Vienna, Austria).

## Results

The mean age was similar for men and women. Women generally had higher levels of education; they were more likely to be divorced, separated, widowed or single, worked fewer hours per week and more often in day shifts. The frequency of self-reported chronic diseases was lower among women. Similar proportions of men and women reported having children under 5 years of age and having a maid (Table 1). Over 90 % of women vs 70 % of men worked in non-manual jobs. The occupational nature of a participant’s present job (classified into four levels: routine/manual; non-routine/manual; routine/non-manual; and non-routine/non-manual) were strongly correlated with educational level for both women and men (Spearman’s correlation coefficient 0.68 and 0.76, respectively, *p* < 0.001) (data not shown).

**Table 1:** Prevalence of suboptimal self-rated health among men and women, by covariables, ELSA-Brasil baseline (2008–10; *n* = 12,017)

	Men (*n* = 5735)		Women (*n* = 6282)	
	All	Suboptimal health (*n* = 1070)	All	Suboptimal health (*n* = 1156)
Age, mean (SD)	49.5 (7.4)	51.1 (6.9)***	48.9 (7.1)	50.1 (7.0)***
Educational level (%)				
University degree	49.6	12.3***	55.6	12.1***
Complete high school	35.4	20.1	37.2	24.0
Complete elementary school	7.9	30.6	4.5	35.8
Never attender or incomplete elementary school	7.1	45.3	2.8	45.2
Marital status (%)				
Married/living together	81.1	19.0**	55.1	17.6***
Divorced/separated/widowers	13.4	19.4	30.8	21.3
Single	5.6	14.4	14.1	15.1
Children under 5 years				
Yes	13.2	17.5	10.0	20.0
No	86.8	18.8	90.0	18.2
Presence of a maid				
Yes	22.2	13.6***	25.5	12.3***
No	77.8	20.1	74.5	20.5
Working hours				
<39	12.8	25.4***	22.3	24.4***
40 to 59	72.0	18.4	67.8	16.9
≥ 60	15.2	14.4	9.9	14.8
Type of shift work (%)				
Daytime	65.9	18.7	65.6	18.4
Daytime shifts	12.5	17.6	17.3	17.3
Mixed or nightshifts	21.6	19.8	17.1	19.9
Presence of self-reported chronic diseases^a^				
Yes	36.4	32.4***	30.9	31.7***
No	63.6	10.8	69.1	12.5

*significant at *p* ≤ 0.05; **significant at *p* ≤ 0.005; ***significant at *p* ≤ 0.001 in chi-square test or t test

^a^The self-reported chronic diseases used in this analysis are the following: hypertension, diabetes, myocardial infarction, stroke and heart failure

The overall prevalence of suboptimal self-rated health was 18.7 % and comparable in men and women (18.7 and 18.4 %, respectively; *p* = 0.72). Men and women who reported suboptimal health were older, had lower educational levels and worked fewer hours per week than those with good self-rated health. Higher levels of suboptimal health were observed in divorced, separated or widowed respondents, those who had no maid and those who reported chronic diseases. Type of work shift and presence of children under 5 years of age were not associated with higher levels of suboptimal health (*p* > 0.05) (Table 1).

In general, women reported frequent work–family conflicts more often than men. For both genders, participants with higher education reported work–family conflict more often. An exception was observed for family-to-work conflict, which was similar for both genders with those with lower education commonly reporting more frequent conflict. In men, frequent family-to-work conflict was associated with a higher level of suboptimal health (*p* < 0.001). For women, the same tendency was observed for three out of four work–family conflict indicators: *work-to-family strain-based, family-to-work conflict* and *lack of time for leisure and personal care* (Table 2).

**Table 2:** Work–family conflict indicators by education level and prevalence of suboptimal self-rated health among men and women; ELSA-Brasil baseline (2008–10; *n* = 12,017)

	Men (*n* = 5735)				Women (*n* = 6282)			
	All	Low educated (*n* = 2888)	High educated (*n* = 2847)	Suboptimal health (*n* = 1070)	All	Low educated (*n* = 2788)	High educated (3494)	Suboptimal health (*n* = 1156)
Work-to-family time-based								
Never to rarely	41.8	48.1	35.4***	19.3	39.8	47.8	33.3***	18.9
Sometimes	32.3	31.4	33.2	17.8	28.2	26.4	29.7	17.4
Frequently	25.9	20.5	31.4	18.7	32.0	25.8	36.9	18.6
Work-to-family strain-based								
Never to rarely	54.3	61.2	47.3***	18.4	45.4	53.9	38.7***	17.9***
Sometimes	29.7	26.4	33.2	17.9	29.6	25.6	32.8	16.8
Frequently	16.0	12.5	19.5	21.1	25.0	20.5	28.5	21.2
Family-to-work								
Never to rarely	67.0	70.2	63.8***	17.4***	68.6	70.2	65.5***	17.6**
Sometimes	25.5	21.0	30.1	19.4	25.5	22.4	28.2	19.0
Frequently	7.5	8.8	6.1	27.0	6.8	7.4	6.4	23.8
Lack of time for leisure and personal care								
Never to rarely	44.2	54.2	34.0***	18.4	32.9	42.5	25.3***	19.3***
Sometimes	32.0	30.2	33.8	18.3	32.6	30.0	34.7	15.6
Frequently	23.8	15.6	32.1	19.7	34.5	27.5	40.1	20.2

*significant at *p* ≤ 0.05; **significant at ≤0.005; ***significant at *p* ≤ 0.001 in chi-square test

In men, the crude analyses showed frequent family-to-work conflict was associated with greater odds of suboptimal self-reported health (OR 1.75; 95 % CI 1.39–2.20; Table 3). After adjustment for covariates, all work–family conflict indicators were associated with suboptimal self-rated health, in a dose–response gradient. This gradient was statistically significant in the case of the family-to-work and lack of time for leisure and personal care indicators. Higher frequency of conflicts in those domains gave increased chances of suboptimal self-reported health. Adjustment by age and education had a mild effect on the association. Working hours and presence of disease showed the highest influence on the association (Table 3). The interaction terms by chi-square test indicated no influence of educational level on the association between work–family conflict indicators and suboptimal self-rated health among men (*p* > 0.10).

**Table 3:** Crude and adjusted odds ratios of the association between work–family conflict indicators and suboptimal self-rated health among men, ELSA-Brasil, baseline (2008–10)

Work-family conflict indicators	OR (CI 95%)				
	Model 1	Model 2	Model 3	Model 4	Model 5
Work to family time-based					
Never to rarely	1.00	1.00	1.00	1.00	1.00
Sometimes	0.91 (0.78–1.06)	0.91 (0.78–1.07)	0.98 (0.84–1.15)	1.03 (0.88–1.21)	1.07 (0.90–1.26)
Frequently	0.96 (0.82–1.14)	0.97 (0.82–1.14)	1.14 (0.96–1.35)	1.28 (1.07–1.53)	1.36 (1.13–1.64)
*AIC*	*5524.1*	*5468.4*	*5305.8*	*5289.9*	*4989.0*
Work to family strain-based					
Never to rarely	1.00	1.00	1.00	1.00	1.00
Sometimes	0.97 (0.83–1.13)	0.98 (0.84–1.15)	1.10 (0.94–1.29)	1.15 (0.98–1.35)	1.18 (1.00–1.39)
Frequently	1.19 (0.99–1.42)	1.21 (1.01–1.46)	1.43 (1.19–1.73)	1.62 (1.33–1.97)	1.67 (1.36–2.05)
*AIC*	*5521.3*	*5464.9*	*5295.3*	*5275.5*	*4975.9*
Family to work					
Never to rarely	1.00	1.00	1.00	1.00	1.00
Sometimes	1.14 (0.98–1.33)	1.18 (1.01–1.38)	1.31 (1.12–1.54)	1.33 (1.13–1.56)	1.34 (1.14–1.58)
Frequently	1.75 (1.39–2.20)	1.75 (1.39–2.20)	1.66 (1.31–2.10)	1.70 (1.35–2.16)	1.82 (1.42–2.32)
*AIC*	*5503.3*	*5447.1*	*5284.9*	*5271.7*	*4971.0*
Lack of time for leisure and personal care					
Never to rarely	1.00	1.00	1.00	1.00	1.00
Sometimes	0.99 (0.85–1.16)	1.03 (0.88–1.21)	1.18 (1.01–1.39)	1.23 (1.05–1.44)	1.25 (1.06–1.47)
Frequently	1.09 (0.92–1.29)	1.14 (0.97–1.35)	1.53 (1.28–1.82)	1.71 (1.42–2.05)	1.77 (1.47–2.14)
*AIC*	*5524.3*	*5467.3*	*5287.2*	*5265.3*	*4965.1*

Model 1: unadjusted. Model 2: adjusted by age, model 3: Model 2 + educational level; Model 4: Model 3 + working hours; Model 5: Model 4 + presence of self-reported chronic diseases

Higher odds of suboptimal self-reported health in crude analyses were observed for women who reported frequent work-to-family strain-based (OR = 1.24; 95 % CI = 1.06–1.44) or family-to-work (OR = 1.46; 95 % CI = 1.16–1.85) conflict (Table 4). Like men, after adjustment for covariates, women with frequent work–family conflict, as measured by all indicators had greater odds of suboptimal self-reported health, and we also observed a dose–response gradient except in lack of time for leisure and personal care. Adjustment for education showed the highest influence on the association between work–family conflict and suboptimal self-rated health (Table 4, Model 3). In fact, educational level interacted with three out of four work–family conflict indicators among women (*time-based work-to-family conflict p* = 0.08; *strain-based work-to-family conflict p* = 0.07; and *lack of time for leisure and personal care p* < 0.001), but there was no evidence of interaction among women for family-to-work conflict (*p* = 0.26).

**Table 4:** Crude and adjusted odds ratios of the association between work–family conflict indicators and suboptimal self-rated health among women, ELSA-Brasil, baseline (2008–10)

Work-family conflict indicators	OR (CI 95%)					
	Model 1	Model 2	Model 3	Model 4	Model 5	Model 6
Work to family time-based						
Never to rarely	1.00	1.00	1.00	1.00	1.00	1.00
Sometimes	0.90 (0.77–1.06)	0.91 (0.78–1.07)	1.02 (0.87–1.21)	1.03 (0.88–1.21)	1.06 (0.90–1.25)	1.10 (0.93–1.30)
Frequently	0.98 (0.84–1.14)	0.98 (0.85–1.14)	1.18 (1.01–1.38)	1.21 (1.03–1.41)	1.29 (1.09–1.51)	1.30 (1.10–1.53)
*AIC*	*6002.8*	*5964.6*	*5757.3*	*5749.0*	*5743.6*	*5532.8*
Work to family strain-based						
Never to rarely	1.00	1.00	1.00	1.00	1.00	1.00
Sometimes	0.92 (0.79–1.08)	0.95 (0.81–1.11)	1.10 (0.94–1.29) 1.11 (0.94–1.30)	1.14 (0.97–1.34)	1.15 (0.98–1.35)	
Frequently	1.24 (1.06–1.44)	1.27 (1.09–1.48)	1.51 (1.29–1.78)	1.54 (1.31–1.80)	1.63 (1.38–1.92)	1.62 (1.37–1.92)
*AIC*	*5992.6*	*5953.3*	*5736.2*	*5727.3*	*5719.5*	*5511.1*
Family to work						
Never to rarely	1.00	1.00	1.00	1.00	1.00	1.00
Sometimes	1.10 (0.95–1.27)	1.13 (0.97–1.31)	1.22 (1.05–1.42)	1.23 (1.06–1.44)	1.24 (1.06–1.44)	1.23 (1.05–1.44)
Frequently	1.46 (1.16–1.85)	1.48 (1.17–1.87)	1.46 (1.15–1.86)	1.47 (1.16–1.88)	1.47 (1.16–1.88)	1.53 (1.19–1.96)
*AIC*	*5994.5*	*5954.9*	*5748.9*	*5740.6*	*5738.7*	*5527.3*
Lack of leisure time and personal care						
Never to rarely	1.00	1.00	1.00	1.00	1.00	1.00
Sometimes	0.77 (0.65–0.90)	0.79 (0.67–0.93)	0.92 (0.78–1.09) 0.94 (0.79–1.11)	0.95 (0.81–1.13)	0.98 (0.83–1.16)	
Frequently	1.05 (0.91–1.23)	1.10 (0.95–1.29)	1.39 (1.18–1.62)	1.42 (1.21–1.67)	1.49 (1.27–1.76)	1.55 (1.31–1.83)
*AIC*	*5987.5*	*5948.4*	*5734.8*	*5724.8*	*5718.3*	*5506.1*

Model 1: unadjusted. Model 2: adjusted by age, model 3: Model 2 + educational level; Model 4: Model 3 + presence of maid; Model 5: Model 4 + working hours; Model 6: Model 5 + Presence of self-reported chronic diseases

Table 5 shows the fully adjusted regression models, including a multiplicative interaction term for women. The results show that the association between frequent work–family conflict and suboptimal self-reported health was stronger in women with higher levels of education. For work-to-family time-based conflict, women with higher levels of education had higher odds for suboptimal self-related health (OR = 1.54; 95 % CI = 1.19–1.99) than less educated women (OR = 1.14; 95 % CI = 0.92–1.42). Similarly, for work-to-family strain-based conflict, women with higher and lower levels of education had OR = 1.91 (95 % CI = 1.48–2.47) and OR = 1.40 (95 % CI = 1.12–1.75). For lack of time for leisure and personal care, women with higher and lower educational levels had OR = 2.60 (95 % CI = 1.95–3.47) and OR = 1.11 (95 % CI = 0.90–1.38).

**Table 5:** Full adjusted logistic regression models of the association between work–family conflict indicators and suboptimal self-rated health including multiplicative interaction term (WFC*educational level) among women

Work-family conflict indicators	OR (CI 95%)^a^	
	Low educated (*N* = 2788)	High educated (*N* = 3494)
Work to family time-based		
Never to rarely	1.00	1.00
Sometimes	1.11 (0.90–1.37)	1.12 (0.85–1.47)
Frequently	1.14 (0.92–1.42)	1.54 (1.19–1.99)
Work to family strain-based		
Never to rarely	1.00	1.00
Sometimes	1.19 (0.96–1.47)	1.13 (0.87–1.47)
Frequently	1.40 (1.12–1.75)	1.91 (1.48–2.47)
Lack of leisure time and personal care		
Never to rarely	1.00	1.00
Sometimes	0.92 (0.75–1.14)	1.27 (0.93–1.74)
Frequently	1.11 (0.90–1.38)	2.60 (1.95–3.47)

ELSA-Brasil baseline, 2008–10

^a^Adjusted by age, educational level, presence of maid, working hours and presence of self-reported chronic diseases

## Discussion

Our findings showed that women had a higher prevalence of work–family conflict and lack of time for leisure and personal care than men. Work–family conflict, as measured by the four indicators, was associated with higher odds of suboptimal health in both genders. The association differs by educational level only among women. More educated women with frequent work–family conflict in the work-to-family time-based, work-to-family strain-based and lack of time for leisure and personal care indicators had the highest odds of suboptimal health compared with women with lower levels of education.

### Findings Compared with Other Studies

The women in our study reported fewer hours worked per week, but more frequent work-to-family conflict (both time- and strain-based) and lack of time for leisure and personal care than men. Although men generally spent more hours in paid work and on commuting than women, women tended to have less time available for leisure and personal care, because they spent more time on unpaid duties [51].

Women in our study were also more educated and more likely to be separated, widowed or single than men, which might indicate why they are in the labour market, and also that they could have less social support at home than men. Higher prevalence of work–family conflict among women has also been reported in previous studies [32, 34, 36, 52]. Research shows that the ability to balance work and private life remains problematic, especially for women [36]. Previous studies found that women’s well-being is positively associated with time spent on paid work, and negatively associated with time spent taking care of home duties [11, 53]. Women generally still have more responsibility for household duties, childcare and caring for older relatives, even in countries with gender mainstreaming and family-friendly policies. In a study among Swedish workers [40], multiple demands from work and private life increased the risk of fatigue in both genders, but only women reported that they wanted to reduce their working hours owing to the burden of managing multiple social roles. For cultural reasons, women may feel compelled to direct their energy and time towards the family and men may believe that their primary goal should be to maintain their position at work [40]. When the demands of work and home are combined, women have more responsibilities and work longer hours than men, and consequently experience stress more often [11, 51].

Despite more frequent work-to-family conflict among women, the association between work–family conflict and suboptimal self-rated health was observed in both genders. Similar results have been found in other studies of different populations [31, 32, 34, 36]. As more women participate in the workforce, both men and women need to operate in both work and family domains and to balance the demands of the two in a limited amount of time. Balancing the demands of work and family in modern life is a source of stress in adulthood and could influence the ranking of priorities for women and men and also reduce the time available for themselves, which can affect health and well-being [18, 29].

Some researchers have pointed out the relevance of distinguishing the direction of the conflict; in other words, whether family life interferes with work or vice versa. This increases understanding of the aspects influencing the work–family conflict, and their health consequences [34, 54]. In line with previous findings, our results suggest both work-to-family and family-to-work conflicts affected self-rated health among men and women [34].

Self-rated health is a multidimensional indicator that can reflect not only physical health, but also state of mind [55]. It is possible that work–family conflicts affect both physical and mental health through the stress cascade. Chronic exposure to stress increases the activities of physiological systems causing ‘wear and tear’, referred to as allostatic load, which is associated with stress hormones protecting the body through short-term adaptation. In the long run, however, this causes mental and physical alterations that increase the risk of disease. Exposure to stress can also affect health indirectly by inducing a profile of adverse behaviours, such as smoking, sedentary habits, unhealthy diet and alcohol consumption [56]. Further research is necessary to determine which mechanisms cause the association between work–family conflict and health outcomes.

### Self-Rated Health and Educational Level

We found that work–family conflict was more strongly associated with suboptimal self-rated health in more highly educated women, suggesting that education modifies the association between work–family conflict and self-rated health. This modification effect was not observed in men. Those in higher status occupations are more likely to have high-pressure jobs, flexible working hours and more responsibilities at work [31, 42], and all these characteristics are related to work–family conflict [23]. Workers in routine jobs, however, probably have greater segmentation of work and family roles, and they are more likely to know when work begins and ends, so they can experience lower permeability and flexibility between work and private life [42]. It is also possible that in the work environment of universities and research institutions, women with higher levels of education have jobs with high control and high demands, as well as a high competitive edge and expectations. They may also have extensive working hours with the boundaries between work and private life less clearly defined [57]. The balance between work and personal life reflects changes in the nature of work and workplaces related to general competition and trends [58]. The academic environment is increasingly investing in information technology to improve knowledge creation and distribution. Researchers and teachers are often asked to perform tasks in very short time periods, and as a consequence, work pressure increasingly spills over into their personal life [57, 59]. This situation could be even more critical among women, who generally have less autonomy to manage their time for self-care because they are often more involved with household tasks than men [3, 57]. Previous studies also identified that the combination of high-powered job and family demands appears more challenging for women than men in higher white-collar occupations [27] or with higher educational levels [23]. All these elements could explain our findings that education modified the association between work–family conflict and self-reported health in women, but not men.

Research into work–life balance has mainly been with white-collar populations. The situation may, however, look different among blue-collar workers and those with lower levels of education [42]. As this study shows, work–family conflicts differ among women with lower and higher levels of education. This is an important finding showing that the social determinants are expressed differently in women with different levels of education.

Some specifics of the Brazilian culture might have influenced the results. It is difficult to determine if there is a discourse on work–family conflict in Brazil, which has a long history of gender inequality. If so, it is possible that this discourse has been more assimilated by women with higher levels of education. Lewis et al. [58] stated that the discourse is mostly present in western societies and that ‘developing’ countries have such different cultures that work–family conflict might be present but is difficult to uncover. The continuation of ELSA-Brasil with its longitudinal design, and other cohort studies, would give more insight in the directionality.

### Strengths and Weaknesses of the Study

The main strength of this study is that it is based on detailed data collected from a large sample of current workers as part of the ELSA-Brasil cohort study. The data provided is an excellent opportunity to study work–family conflict in a middle income country where this issue has hardly been investigated. To the best of our knowledge, this is the first large-scale study examining work–family conflict in relation to self-reported health in Brazil. Besides, this study included one indicator that has not been used in work–family conflict research before. This indicator considered the influence of both domains simultaneously (work and family) on lack of time for leisure and personal care. This can be seen as an additional strength because this indicator presents new information about important aspects of modern life and may cast new light on studies of work–family conflict [29]. Our study highlights the importance of examining the interaction effect of educational level and the direction of work-to-family and family-to-work conflict, as well as the effect of both conflicts simultaneously in separate scales. We also investigated the influence of work-to-family conflict by time-based and strain-based conflicts, which have not been the focus of many previous investigations [21, 22]. The behaviour-based conflict was not included in our study; according to some authors [17] few studies have investigated this dimension, and the meaning of behaviour-based conflict needs to be clarified.

Nevertheless, the study also has some limitations. This is a cross-sectional analysis, and temporality cannot be established. The temporal direction of the association is therefore less clear, and the associations may be bidirectional or resulting from common causes. ELSA-Brasil comprises a particular population (civil servants), so it may not be possible to generalize the results. However, the results may reflect the broader population in the same situation, for example, middle class-employed individuals or general civil servants who live in the larger metropolitan areas in Brazil. Moreover, given the nature of our sample, we did not have a substantial variation in terms of job levels, which may be considered a further limitation [60]. Our sample was homogeneous in terms of job tenure, and all participants were salaried employees from the public sector. Even so, our study sample had enough socioeconomic variability to capture gender differences in work–family conflict and the relationship with self-rated health across levels of education.

Although there is extensive research about work–family conflict, there is no consensus on the best way to measure this construct [17]. The literature describes different instruments and high variability in the number of dimensions and items, content, and presence of directionality of conflict [17, 27, 31, 32, 36, 40, 60]. It is possible that the work–family conflict indicators used in this paper only partly represent the whole work–life conflict model because isolated items were used to measure complex dimensions of a higher construct; this is an important limitation of our study. It is, however, an important strength of our study that the work–family conflict indicators are not combined. Including different pathways in a single question, or combining pathways would make it difficult to understand the effect of the work–family conflict indicators on self-rated health. The indicators each show their own connections to self-rated health and the results are limited to the directionality and internal category.

## Conclusion

Our findings are in line with previous research showing an association between work–family conflict and health. More frequent work–family conflict was associated with suboptimal self-rated health by all the work–family conflict indicators tested. We also found that educational level modified these associations, but only among women.

Future research should incorporate the role of cultural differences around gender in a Brazilian context, to show how this affects family and work spheres for both genders. It is also necessary to understand the effect of decision latitude, social support and other relevant moderating effects on the relationship between work–family conflicts and health, according to socioeconomic position and job occupation, and across gender groups. We believe that the opportunity for personal development for both genders and enrichment of everyday family life will be guaranteed by higher gender equality in taking care of home duties and looking after children. This change might decrease stress levels and positively influence priorities for women and men in the use of time for themselves, improving health and well-being. This is especially important in countries like Brazil, where large gender inequalities interact with other social and economic inequalities. Handling the spillover between job and family demands in modern life, especially in big cities, is a great challenge and more than individual (or family) arrangements are necessary. Macro-level and organizational policies are also necessary to promote changes in traditional patterns of behaviour and to foster gender equality and social justice.
